# A quantitative study of nurses perception to advance directive in selected private and public secondary healthcare facilities in Ibadan, Nigeria

**DOI:** 10.1186/s12910-022-00825-5

**Published:** 2022-08-25

**Authors:** Oluwaseyi Emiola Ojedoyin, Ayodele Samuel Jegede

**Affiliations:** University of Ibadan, Ibadan, Nigeria

**Keywords:** Treatment preference, End-of-life care, Living will, General hospital, Private hospital

## Abstract

**Objectives:**

The study evaluated nurses’ perceptions on the benefits, drawbacks, and their roles in initiating and implementing advance directives (AD) at private and public secondary healthcare units.

**Methods:**

The study adopted a cross-sectional, comparative-descriptive research design and was anchored on the structural functional theory. A total of 401 nurses (131 private and 270 public) were chosen on purpose. The data was collected between January and March 2018 among nurses at the selected hospitals. Analysis was done via SPSSv28.0.1.0.

**Results:**

Compared to nurses working in private healthcare facilities (72.5%), the majority of nurses at the public healthcare facilities (75.2%) indicated a more favorable opinion of AD’s benefits and (61.9%) felt they had a substantial involvement in the development and execution of AD than their private counterpart (56.5%). Similarly, 60.7% of nurses employed by the government agreed that AD has some disadvantages compared to those employed by the private sector (58.8%). Significantly, Christian nurses are 0.53 times less likely than Muslims to contest AD’s benefits; 0.78 times less likely than Muslim to disagree that AD has flaws; and 1.30 times more likely than Muslim nurses to deny they contributed to the development and execution of AD, though not significant.

**Conclusion:**

Making decisions at the end-of-life can be challenging, thus AD should be supported across the board in the healthcare industry. Nurses should be trained on their role in developing and implementing AD, as well as on its advantages and how to deal with its challenges.

## Introduction

Humans are born with the fundamental right to life. Due to this, many view death as undesirable, and even healthcare professionals avoid the discussion [1, 2]. However, death is an inevitable, natural occurrence that all patients with life limiting illnesses should be prepared for in order to minimize distress at the end stage of life. Advance care planning (APC) is a method of communicating intentions that allows patients to let their loved ones and healthcare providers know in advance how they would like to be treated. One strategy in APC that aid readiness for future illness-related incapacitation, patients’ autonomy and dignity is advance directive (AD). AD is a written document or spoken declaration that enables competent people to make and document their healthcare decisions in advance [3–6]. Although patient’s “written directives” is a helpful tool for determining their preferences, tradition still dominates in most Africa countries. AD is yet to be legalised in Nigeria [7]. However, patients verbally expressed their preferences of care to healthcare professionals, and some even name individuals to make treatment decisions on their behalf when they are incapacitated [7]. These do not only promote patient participation in EOL discussion but also mitigate the paternalistic aspect of Nigeria’s healthcare system [8, 9].

The Nigeria healthcare unit is divided into 3—primary, secondary and tertiary. Healthcare facilities at each unit can be privately owned or publicly owned. The difference between the two hospitals are found in their governance—the former are owned and run by an individual or group of individuals while the later are managed and funded by the government. The secondary healthcare facilities—which was the focus in this study manage advanced medical conditions [10] and it had been shown that, private hospitals are mostly used by Nigerians [11]. Nurses at these two facilities play significant roles in patients’ care. They provide medical, emotional, educational, patient-centered care and also serve as mediator between patients and doctors [12, 13]. These put them in the best position to help in advance care planning—a procedure for communicating patients’ intentions [14]. Therefore, comparing the viewpoints of these nurses regarding AD will help to determine how end-of-life care is provided at this healthcare unit. There is a paucity of data on nurses’ perceptions of AD in Nigeria, and no study has described nurses’ perceptions at both private and public secondary healthcare facilities to the best of our knowledge. Previous researches focused on patient perception of AD and advocacy for AD inclusion in the country’s healthcare system [5, 7, 15]. This study therefore compared perceptions of nurses at the private and public secondary healthcare facilities on the advantages, roles and shortcoming of ADs in Ibadan, Oyo state, Nigeria.

## Theoretical orientation

### Structural–functional model

A sociological theory known as functionalism views society as an organism of several elements (social institutions) that work together to maintain and reproduce the society [16]. These social institutions are typical means by which a society can attend to and satisfy both its social and individual needs. For instance, hospital is a social institution with many healthcare professionals collaborating to provide the best possible healthcare services to the community. Social institutions are also examined by functionalists in terms of the roles they played. Hence, to comprehend every part of society (e.g. doctor, nurse, teacher, AD, etc.) and how they affect social cohesion, reproduction, or the effective operation of a larger community, the functions of such institutions, beliefs, or ideologies are taken into considerations.

Merton however proposed that not all structure, custom, religion, ideology etc., serves positive purposes because they may serve both manifest and latent functions [17]. The latent functions are elements of behaviour or functions that are not openly declared, recognised, desired or intended. While the manifest functions are elements of conduct or functions that are conscious and purposefully [17]. Both the latent and manifest functions of AD was examined in the present study.

## Methods

### Research design

The study was a cross-sectional comparative-descriptive research design.

### Participants

Nurses working in government-owned (public) and privately-owned secondary healthcare institutions as well as nursing students at the chosen hospitals participated in the survey.

### Study location

The study was carried out in Ibadan, the Oyo state capital of Nigeria. Ibadan was deliberately chosen because it is Nigeria’s third-most populous city after Lagos and Kano, and because the region has historically had limited access to health care services [18]. Six out of eleven local government areas (LGAs) in Ibadan were chosen for this study—Ibadan Northeast, Ibadan Southwest, Ibadan Southeast, Ibadan North, and Egbeda. The high number of secondary health care facilities in these LGA coupled with the fact that no study on AD has been carried out among nurses in these locations were a deciding factors.

### Sampling technique

A convenient non-probability sampling method was used to select nurses. This was employed due to the low staff strength, heavy workload and burnout on available staff. Five general hospitals and ten private secondary hospitals were included in the study—because of the high proportion of private secondary health facilities to public secondary health care facilities in the location and Nigeria as a whole [19]. A total of four hundred and one (401) nurses—270 nurses from public and 131 nurses from private hospitals—participated in the study.

### Research instrument

Questionnaire was used to elicit information from respondents. Data was gathered in 2018 between January and March. The surveys were distributed to all nurses on-duty at their offices. A total of 430 survey was distributed out of which 401 was returned, making a 93% response rate. A total of 7% of the data was missing because several nurses worked night shifts, took the survey home, went on leave, and neglected to return the questionnaire.

The survey questions were developed after careful examination of literature from various countries [20–23]. Additionally, the opinions of three experts on prospective contents that required evaluation were sought. The questions’ ambiguity, relevance, clarity, and comprehensiveness were also evaluated. They assessed the questionnaire’s validity in terms of both face and content. The comments was examined, and the changes were added in the final survey. However, pilot survey was not conducted.

The questionnaire comprises two sections. The first section was on respondents’ socio-demographical characteristics. The second section was on perception and comprises 13 items—4 questions on benefits of AD, 5 questions on nurses’ roles in the initiation and implementation of AD, and 4 questions on shortcomings of AD. A 5-point likert scale was used to grade the responses of the participants ranging from strongly agree (5) to strongly disagree (0).

### Data analysis

Data entry, cleaning, and analysis were performed using SPSS 28.0.1.0. Descriptive statistics was calculated for the socio-demographic and perception of nurses to AD. For questions on benefit of and nurses role in AD initiation and implementation, strongly agree and agree responses were merged to form correct perception to AD while, neutral, disagree and strongly disagree was merged as incorrect response. For questions on shortcomings of AD, strongly agree, agree and neutral responses were merged to form incorrect perception to AD while, disagree and strongly disagree was merged as correct response. The score for a correct response was two, while the score for an incorrect response was zero. The mean was calculated and response below the mean was considered as negative perceptions and those above or within the mean as positive perception.

On both the total benefits and drawbacks questions, 75% percentile (scoring three or more out of the four questions) was defined as positive perception, while 25% percentile (scored one out of the four questions) was labeled as negative view. The percentiles for the role of nurses in the initiation and implementation of AD were 60% (scoring 3 or more out of the 5 questions) and 40% (scored 2 or fewer out of the 5 questions). Differences between public and private nurses and nurses religion was examine using the odd ratios.

Reliability assessment of the questionnaire was conducted using Cronbach’s alpha coefficient based on Heden scale as cited in Peicus et al. [21] internal reliability assessment and recommendation. It stated that, a scale is reliable if the Cronbach’s alpha is > 5. The Cronbach alpha for the study is (0.62).

### Ethical consideration

The Oyo State Research Ethics Review Committee, with reference number AD13/479/837, as well as administrative officers from each of the chosen hospitals and each participant, gave their approval before the data collection began.

## Result

### Characteristic and representative of nurses in the study

The complete list of participants characteristics is shown in Table 1 below. The majority of respondents are women (88.9% public and 96.9% private). The majority (56.7%) of staff members at public hospitals hold diploma degrees, with one (0.4%) PhD degree holder. In contrast to the government hospitals, where 44.1% of participants had more than ten years of work experience, more than half (55.7%) of the private participants are within 1–5 years of work experience group. Predominant group are Yoruba (94%), Christians (79.3%) and more respondents from the public hospital (67.3%).

**Table 1:** Descriptive characteristics of participant nurses (N = 401)

Variable	Category	Type of institution	
		Public frequency (%)	Private frequency (%)
Gender	Male	30 (11.1)	4 (3.1)
	Female	240 (88.9)	127 (96.9)
Educational qualification	Diploma	153 (56.7)	57 (43.5)
	Bachelor	91 (33.7)	72 (55)
	Master	25 (9.3)	2 (1.5)
	Doctorate	1 (0.4)	0 (0)
Work experience	< 1 year	30 (11.1)	13 (9.9)
	1–5 years	75 (27.8)	73 (55.7)
	6–10 years	52 (19.3)	22 (16.8)
	> 10 years	113 (41.9)	23 (17.6)
Current position	Student	14 (5.2)	1 (0.8)
	Nursing officer	115 (42.6)	111 (84.7)
	Senior nursing office	47 (17.4)	11 (8.4)
	Principal nursing officer	42 (15.6)	1 (0.8)
	Asst chief nursing officer	23 (8.5)	5 (3.8)
	Chief nursing officer	29 (10.7)	2 (1.5)
Ethnicity	Yoruba	255 (94.4)	122 (93.1)
	Igbo	9 (3.3)	7 (5.3)
	Hausa	2 (0.7)	0 (0)
	Other	4 (1.5)	2 (1.5)
Religion	Christianity	204 (75.6)	114 (87)
	Islam	66 (24.4)	17 (13)
	Traditional	0 (0)	

### Distribution of nurses perception of benefits of advance directive

As shown in Table 2 below, most of the nurses agreed the AD is helpful when deciding how to treat patients (public-94.5% and private-93.1%); makes decision easier (public-88.2%, private-93.1%), minimize family conflict (public-85.9%, private-80.1%) and majority felt it reduced wasteful spending (public 77%, private 77%);

**Table 2:** Frequency and percentage distribution on nurses perception on benefits, nurses roles and shortcomings of advance directive

Variable	Perception rank	Type of institution	
		Public (%)	Private (%)
*Benefit of AD*			
AD are useful for Professionals when making medical decision	Agree	94.5	93.1
	Neutral	3.3	4.6
	Disagree	2.2	2.3
Widespread use of AD will help to contain unnecessary medical expenditures	Agree	77.0	77
	Neutral	15.6	13
	Disagree	7.4	10
A patient representative in the durable power of Attorney makes decisions easier for health professionals in those situations where the patients cannot express themselves	Agree	88.2	93.1
	Neutral	6.3	3.1
	Disagree	5.5	3.8
AD reduce family discord	Agree	85.9	80.1
	Neutral	5.2	15.3
	Disagree	8.9	4.6
*Roles of nurses in AD*			
Nurses are best suited to provide information about advance directives to the patient and their family members	Agree	78.1	73.3
	Neutral	10.7	16.8
	Disagree	11.1	9.9
Nurses are unique in accessing appropriate time for AD discussion	Agree	84.4	78.6
	Neutral	8.5	15.3
	Disagree	7.1	6.1
Nurses should initiate end of life discussions	Agree	75.9	57.3
	Neutral	9.3	20.6
	Disagree	14.8	22.1
Nurses who disagree with patient directive can refer the case to another nurse	Agree	56.7	67.9
	Neutral	23.0	21.4
	Disagree	20.4	10.7
Nurses confer with physician when patient will has not been honored	Agree	74.1	77.0
	Neutral	14.1	15.3
	Disagree	11.9	7.7
*Shortcomings of AD*			
AD can be difficult to interpret	Agree	37.0	32.1
	Neutral	20.7	32.0
	Disagree	42.2	35.9
AD can be uncertain	Agree	51.8	39.7
	Neutral	19.6	28.2
	Disagree	28.6	32.1
AD may request provision of treatment not best for patient	Agree	55.6	65.7
	Neutral	13.0	11.5
	Disagree	31.4	22.9
AD may not represent the current wishes of the patient	Agree	57.8	52.6
	Neutral	18.1	17.6
	Disagree	24.1	29.8

### Perceived nurses role in advance directives

Majority agreed that nurses are crucial in educating about AD (public-78.1%, private-73.3%); in best position to access the appropriate time for end-of-life discussions (public-84.4%; private-78.6%) and are responsible to initiate end-of-life discussion (public-76%, private-57.3%). More participants in the private facilities than those at the public agreed that nurse can transfer a patient to another nurse when not comfortable with the directives.

### Perceived shortcomings of advance directive

More participants in public (42.2%) than private (35.9%) disagreed that interpreting AD can be challenging. Two-thirds of private nurses (65.7%) and 55.5% of nurses in the public hospital agreed that AD can lead to requests for care not in the patient’s best interests. The little more than half of the participants felt AD might not accurately reflect patient’s current preferences (public-57.8%; private-52.7%) and uncertain (public-51.8%, private 39.7).

### Classification of nurses responses into positive and negative perception

Table 3 shows how nurses generally perceived the benefits of AD, their involvement in its initiation and execution, and its perceived drawbacks. Majority of nurses in the public sector (75.2%) and private sector (72.5%) agreed AD is beneficial to patients, their families, and healthcare providers. More participants in the public sector (61.9%) than private (56.5%) thought they played a critical role in the development and implementation of AD. More nurses (60.7%) in the public sector concurred that AD had drawbacks than its private counterpart (58.8%).

**Table 3:** Classification of nurses total scores on perception to AD

Perception ranks to advance directive	Type of hospital type	
	Public N (%)	Private N (%)
*Perceived benefit of AD*		
Positive	203 (75.2)	95 (72.5)
Negative	67 (24.8)	36 (27.5)
*Perceived nurses roles in AD*		
Positive	167 (61.9)	74 (56.5)
Negative	103 (38.1)	57 (43.5)
*Perceived shortcoming of AD*		
Positive	106 (39.3)	54 (41.2)
Negative	164 (60.7)	77 (58.8)

### Differences on nurses perception to advance directive

Table 4 below displays how Muslim nurses and Christian nurses perceive AD using odd ratios. Significantly, Christian nurses are 0.53 times less likely than Muslims to contest AD’s benefits; are 0.78 times less likely than Muslim to disagree that AD has flaws but are 1.30 times more likely than Muslim nurses to deny they contributed to the development and execution of AD, albeit, these differences are not statistically significant.

**Table 4:** Odd ratio on nurses’ perceptions of benefit, their roles in the initiation and implementation and shortcomings of AD

	OR	95% CL
*Benefit of AD*		
Religion (Christian/Muslim)	0.53	0.321 to 0.86
Disagreed	0.736	0.59 to 0.91
Agreed	1.40	1.06 to1.86
*Nurses role in AD*		
Religion (Christian/Muslim)	1.30	0.79 to 2.19
Disagreed	1.18	0.86 to 1.63
Agreed	0.90	0.75 to 1.09
*Shortcomings of AD*		
Religion (Christian/Muslim)	0.78	0.48 to 1.27
Disagreed	0.86	0.64 to 1.15
Agreed	1.10	0.90 to 1.35

## Discussion

This study focused on nurses’ perceptions on the benefits, the role of nurses, and the negative aspects of AD at public and private secondary healthcare units in Ibadan, Nigeria. Positive perception regarding AD advantages was found among nurses at both public and private secondary healthcare units. This supported previous reported finding in Australia and Korea. According to these researches, AD guarantee patient autonomy, improve end-of-life care, and give patients a chance to reflect on their own dying stage and demise [22–24]. The study findings also agreed with prior researches where it was reported that the enforcement of ADs relieved families and patients’ financial, emotional weariness and disagreement [24, 25] as we found that, participants agreed that AD can reduced needless stress, excessive spending and prevented or resolved conflict among healthcare practitioners, patients and patients relatives.

Nurses are more available at hospital and are closer to patients than any other healthcare practitioners. As a result, they agreed they are the best resource for patients and their families seeking information about AD. This support earlier researches in Portugal, Korea, New Zealand, and Australia [3, 12, 22, 24, 26]. The disparity reported on who is proficient in figuring out the appropriate time to initiate AD among the two group of nurses could be attributed to the quantity and quality of training enjoyed by these nurses. While more trainings are planned for nurses at the public sector, little of such training is available for nurses at the private sector in Nigeria. Davidson et al. also reported that nurses are in the best position to initiate AD [12]. On who should start the end-of-life conversation with a patient, the nurses at the two healthcare facilities had contrasting opinions. Nurses at private facilities saw it as the doctors’ obligation to begin and record the decision while they made the document readily available when needed, in contrast to nurses at public hospitals who saw it as their role. These was similar to findings in South Africa, Korea and Australia by Bull and Mash, Son et al., and Hobden et al. [24, 27, 28], where nurses saw themselves as the custodians of AD document rather than its initiators and/or implementers. The findings demonstrated that nurses in the private sector are more likely to refer patients whose orders they find objectionable to another nurse or facility. These both supports Siamak’s [23] findings that nurses have the autonomy to decline participation in the withdrawing or withholding of treatment if such a decision contradicts their personal and/or professional convictions and Hobden’s [27] findings where 60% of their study participants showed neutrality or disagreement that ADs will still be adhered to even if the medical team does not agree with them. Fear of litigation and the fact that nurses at the public sector enjoyed more autonomy, employment security, and public reputation than those in the private sector are some contributing factors to this [26]. Making known and reporting violation of patients’ directives were found to be nurses’ responsibilities in the present study. This was in consistent with Hobden et al. [27] that found nurses play a key role in ensuring that patients’ preferences are honored throughout end-of-life care.

Regarding AD’s shortcomings, the study demonstrates consensus that AD has some degree of negativity, but to various degrees. Over 50% of the study participants in the two sectors agreed and are neutral on the statement that many ambiguous terms are frequently used in AD without enough context or justification thereby making it difficult to interpret. Previous researchers have also noted that unclear instructions and the use of ambiguous language could lead to misreading of patients’ preferences [20, 21, 27, 29]. The fact that patients’ mostly give their directives verbally when they are critically ill and sometime by their relatives in Nigeria can also contribute to the misapprehension of the directives [7, 30]. More public sector nurses thought it was challenging to prove that AD is certain and accurately reflect patients’ current preferences and this made its implementation challenging. Reasons could be because, patients’ decisions regarding their treatment preference evolved over the illness episode due to factors like finance, relative decision, religious beliefs among others. However, these changes may not have reflected in the patient AD or known to the patient proxy. These contributed to the controversy in its implementation. Thus, decisional conflict that results from translating a written order into practice has previously been identified as an obstacle to the application of AD [26–28]. Ernestina et al. [3] showed that AD can fail in practice if changes in patient personal value fail to reflect in the directive. Therefore, AD should be periodically addressed and revisited for timely updates [21]. More than half of the study participants agreed that, there are chances that patients will asked for treatment that is not in their best interests in their AD. This finding supported researches conducted in Queensland, Australia, and Korea where it was reported that AD inhibited medical personnel from providing ethically and medically appropriate treatment to patient [20, 26, 29]. Inadequate knowledge and wrong cultural preconceptions about health, illness and treatment among patients could contributed to this perception. This study has been able to support existing knowledge that religion affiliation influence perception to end-of-life care [31, 32]. While more Christian nurses are optimistic on the benefits of AD than Muslim nurses, more Muslim nurses believed they have a role to play in its initiation and execution than their Christians counterpart and thought AD had lesser flaws than the Christians. One of the tenets of Islam is to work for this life as if you were going to live forever and strive for the afterlife as if you were going to die tomorrow [33]. The Holy Qur’an also instructs Muslims to prepare and strategize their affairs. These may have influenced their perception that they have a greater role to play in the planning and implementation of the patient’s AD and support for AD. The Christian religion also supports AD as useful because it aids patients to avoid unbeneficial treatment [34].

In line with the theoretical explanation, the study had demonstrated that although AD has some benefits, such as quick decision-making, conflict resolution, and the prevention of wasteful spending; nurses as members of the healthcare team have a role to play in its initiation and implementation of AD. However, AD does have certain unintended consequences, which are its drawbacks [17].

This study has added to the corpus of research by identifying the perception of AD at the secondary healthcare facility in Nigeria and the chance that it will be adopted by nurses, who make up the majority of healthcare professionals. The study is limited by the use of the Likert scale to score nurses’ perceptions, which might have inhibited participants from fully expressing their perspectives on the matter. Further research should look into the acceptance of AD among terminally ill patients and their families as well as the amount of abuse or improper inducement of AD among healthcare professionals in secondary and tertiary healthcare facilities in Nigeria.

## Conclusion

Making decisions in the final stages of life might be challenging, however AD may make these challenges easier. As a result, AD should be acknowledged in all healthcare sectors as a tool capable of granting patients’ liberty and dignity. Both in the public secondary healthcare unit and the private unit, nurses play a vital role as care providers in the development and execution of patient Ads. However, some of the difficulties in implementing AD that have been identified in this study should be addressed by stakeholders, and nurses at both sectors should be provided with necessary training on how to avoid these difficulties.

## Data Availability

Due to confidentiality rules, the datasets created and/or analyzed for the current work are not publically accessible, but they are available from the corresponding author upon justifiable request.
